# Delirium prevalence and delirium literacy across Italian hospital wards: a secondary analysis of data from the World Delirium Awareness Day 2023

**DOI:** 10.1007/s41999-024-01019-5

**Published:** 2024-07-18

**Authors:** Alice Margherita Ornago, Elena Pinardi, Chukwuma Okoye, Paolo Mazzola, Maria Cristina Ferrara, Alberto Finazzi, Peter Nydahl, Rebecca von Haken, Heidi Lindroth, Keibun Liu, Alessandro Morandi, Giuseppe Bellelli, National Collaborators, Adela Goldberg, Gideon Caplan, Magdalena Hoffmann, Ricardo Kenji Nawa, Thiago Silva, Roberta Esteves Vieira de Castro, Karla Krewulak, Tanya Mailhot, Kirsten Fiest, Marie Oexenbull, Tej Pandya, Metaxia Papanikolaou, Julie Benbenishty, Shelly Asheknazy, Mohan Gurjar, Suzanne Timmons, Chi Ryang Chung, Muhammed Elhadi, Mark van den Boogaard, Hilde Woien, Maria Carolina Paulino, Gabi Heras, Abdullah M. Alhammad, Dejan Markovic, Yie Hui Lau, Terry Quinn, Gabi Heras, Carola Gimenez-Esparza Vich, Marie-Madlen Jeitziner, Matthias Exl, Bronagh Blackwood, E. Oh, M. Fuchita, K. Maya, K. Smith, T. Brynes, J. Palakashappa, S. Cotton, B. Hetland, N. McAndrew, M. Mulkey, Italian Participating Clinicians, Clara Agostino, Yanely Sarduy Alonso, Ilaria Bandera, Costanza Berti Ceroni, Filippo Binda, Mario Bo, Maria-Cristina Bragaglia, Enrico Brunetti, Luca Bucciarelli, Stefano Cacciatore, Monica Cadei, Gianni Casella, Martina Cavara, Incoronata Chiusolo, Antonio Ciambrone, Giulia Clericò, Alessandra Coin, Marika Colacicco, Alessandro Di Risio, Valter Favero, Paola Claudia Fazio, Rosa Filippelli, Luigi Francioni, Alessandro Galazzi, Barbara Gamba, Giordana Gava, Simona Gentile, Emma Giovannini, Barbara Guadi, Mary Jane Isidro, Angela Iurlaro, Maria Legierska, Silvia LoMele, Vitalba Maniscalco, Michela Marca, Valentina Martella, Claudia Massaro, Marialaura Matacena, Elena Merli, Giuseppina Migliorino, Alessandro Monesi, Valeria Nativio, Giulia Principato, Daniela Quattrocchi, Daniela Perelli Ercolini, Sabina Perelli Ercolini, Francesca Paternoster, Lorenzo Pilati, Samanta Pittarello, Roberto Presta, Daniela Petronela Radeanu, Giulia Ratto, Carla Recupero, Alessandro Reggiani, Anna Rita Reggiani, Antonella Risoli, Barbara Romagnoli, Francesca Ruma Romana, Loretta Ruggeri, Francesco Salis, Elena Trotta, Salvatore Tupputi, Edoardo Varatta, Valentina Viani, Maria Beatrice Zazzara

**Affiliations:** 1 School of Medicine and Surgery, University of Milano-Bicocca, Piazza Dell’Ateneo Nuovo 1, Milan, Italy; 2 Aging Research Center, Department of Neurobiology, Care Sciences and Society, Karolinska Institutet and Stockholm University, Stockholm, Sweden; 3 Acute Geriatric Unit, IRCCS San Gerardo Foundation, Monza, Italy; 4 Nursing Research, University Hospital Schleswig-Holstein, Kiel, Germany; 5 Institute of Nursing Science and Development, Paracelsus Medical University, Salzburg, Austria; 6 Department of Anesthesiology, University Hospital Mannheim, Mannheim, Germany; 7 Division of Nursing Research, Department of Nursing, Mayo Clinic, Rochester, MN USA; 8 Center for Aging Research, Regenstrief Institute, School of Medicine, Indiana University, Indianapolis, IN USA; 9 Critical Care Research Group, The Prince Charles Hospital, Brisbane, Australia; 10 Department Intensive Care, Radboud University Medica Center, Nijmegen, The Netherlands; 11 Intermediate Care and Rehabilitation, Azienda Speciale Cremona Solidale, Cremona, Italy; 12 Parc Sanitari Pere Virgili, Val d’Hebron Institute of Research, Barcelona, Spain

**Keywords:** Delirium, Hospital, Survey, Quality improvement

## Abstract

**Purpose:**

Delirium, a common medical emergency among hospitalized patients, requires effective detection and management protocols. This study aims to evaluate the delirium point prevalence and its management across Italian hospitals, categorized by delirium literacy levels. Additionally, it seeks to identify prevailing barriers and future priorities in delirium practice and research.

**Methods:**

We analyzed data from World Delirium Awareness Day (WDAD) on March 15th, 2023, collected by participating clinicians in Italian hospitals. High delirium literacy (HL) was determined based on the use of validated delirium assessment tools and the presence of a written protocol for delirium management. Conversely, low delirium literacy (LL) was determined by meeting only one or neither of these criteria.

**Results:**

Fifty-eight hospital wards participated in the survey, with 25 (43.1%) classified as HL. The overall reported point prevalence of delirium was found to be approximately 10%. Notably, the reported prevalence was twice as high in the HL group compared to the LL group. Despite minimal differences compared to the other group, the HL group demonstrated greater adherence to appropriate delirium management strategies, encompassing both non-pharmacological and pharmacological strategies. Critical gaps in delirium care emerged, including suboptimal management practices, barriers to implementing evidence-based strategies, and insufficient awareness and training among professionals.

**Conclusion:**

The study highlights the suboptimal identification and management of delirium among Italian hospitals, emphasizing the necessity of enhancing awareness and implementing evidence-based strategies. Addressing these shortcomings is crucial for optimizing delirium care, improving patient outcomes, and mitigating the burden of delirium in hospital settings.

**Supplementary Information:**

The online version contains supplementary material available at 10.1007/s41999-024-01019-5.

## Key summary points

**Aim:**

To assess the point prevalence of delirium and its management across Italian hospitals, according to delirium literacy levels, pinpointing prevailing barriers and future priorities in delirium practice and research.

**Findings:**

Critical gaps in delirium care were identified, including suboptimal management practices, barriers to evidence-based medicine implementation, and insufficient awareness and training among healthcare professionals.

**Message:**

Enhanced awareness and adoption of evidence-based strategies for delirium are essential for optimizing delirium care, improving patient outcomes, and alleviating the burden of delirium in hospital settings.

**Supplementary Information:**

The online version contains supplementary material available at 10.1007/s41999-024-01019-5.

## Introduction

Delirium, a neuropsychiatric syndrome characterized by an abrupt onset and fluctuating disruption in consciousness, attention, and cognitive function [1], poses a significant challenge across various clinical settings. Its incidence and prevalence vary considerably, depending on the context and patient demographics [2, 3]. Delirium is relatively uncommon in community dwellers and outpatients, whereas it is more frequent in individuals with acute and exacerbated chronic illnesses [4, 5]. Consistent evidence shows that, on average, one in five hospitalized patients aged 65 years and above experience delirium daily, regardless of the hospital ward type [6, 7].

Delirium occurrence is independently associated with several adverse outcomes, including prolonged hospital stays, increased vulnerability to complications (e.g. pressure ulcers, incontinence, and falls), high mortality rates, and impaired physical and cognitive recovery [8, 9]. Consequently, it also carries substantial implications for healthcare expenditures [10, 11]. Furthermore, as the likelihood of adverse outcomes increases with delay in delirium diagnosis [12], the critical importance of early detection and proactive management strategies is evident.

Current primary management approaches encompass the utilization of validated screening tools and multidomain interventions targeting precipitating conditions, medication review, distress management, complications mitigation, and addressing environmental factors to sustain patient engagement [13–15]. Despite their well-documented effectiveness [16], integrating these strategies into acute care settings has proven challenging for healthcare organizations [17, 18]. Key barriers to successful integration include time and staffing constraints, inadequate multi-professional collaboration, and insufficient knowledge among personnel [19]. These barriers contribute to the lack of routine screening for delirium and, consequently, its suboptimal management.

In Italy, there is a notable gap in understanding the extent to which healthcare centers incorporate evidence-based protocols for preventing, diagnosing, and treating delirium into daily clinical practice. This gap is particularly concerning due to the adverse prognostic implication of delirium, compounded by its prevalence. Previous nationwide studies have reported a delirium point prevalence of 22% and an elevated risk of short-term mortality among hospitalized older persons with delirium [3, 9], suggesting that detection and appropriate management of this condition should be a priority for healthcare systems.

We hypothesize that the attitude to delirium screening and implementing appropriate prevention and management strategies within hospital wards may be influenced by their level of delirium knowledge and understanding (i.e. delirium literacy).

Therefore, this study aims to assess the reported point prevalence of delirium and explore management strategies based on delirium literacy levels across Italian hospitals. Furthermore, it seeks to identify current perceived barriers and future priorities in delirium practice and research.

## Methods

This study is a secondary analysis of Italian data derived from a global delirium prevalence study on World Delirium Awareness Day (WDAD) on March 15th, 2023.

Ethical approval for the study was obtained from the Institutional Review Board of the University Mannheim (2022–617) and registration was completed with the German Clinical Trials Register (DRKS00030002, https://drks.de/search/de/trial/DRKS00030002).

A request for participation has been disseminated through social media platforms, professional networks, and personal contacts. National coordinators were responsible for recruiting clinicians and distributing the survey on the specified study day. All participating clinicians provided informed consent for the research at the outset of the questionnaire, which was administered online via SurveyMonkey [20].

### Survey content

The questionnaire comprised 39 questions divided into fourteen sections. The first six sections covered data protection and consent, as well as the demographics of the professionals completing the survey. Additionally, these sections collected hospital and ward/department-specific data. The other sections covered data related to delirium assessment, structure, and process, focusing on management and implementation strategies, barriers, and perspectives. Delirium point-prevalence was evaluated both at 8 a.m. and 8 p.m. The respondents were instructed not to directly assess the presence of delirium but to report the assessment method used, the number of patients in the ward/unit at each time point, and the number of patients with and without delirium identified by ward/unit personnel. Importantly, no patient-level sensitive information was collected.

Further details on study design, preparation, inclusion and exclusion criteria, and data collection procedures have been already described elsewhere [21].

### Sample characteristics

For study purposes, starting from 112 completed unique national surveys we initially excluded those from long-term care settings (e.g., rehabilitation, nursing home, intermediate care; n = 25). Subsequently, surveys from ICU and high acuity units were also excluded (n = 29) to maintain consistency in examining delirium within non-intensive care settings.

### Delirium literacy levels

Delirium literacy levels were determined based on two criteria: (i) the routine utilization of a validated delirium assessment tool and (ii) the presence of a written protocol for delirium management. The former aspect was ascertained by assessing whether the tool had been acknowledged in the literature as reliable and validated [22]. High delirium literacy (HL) was defined by the fulfillment of both criteria simultaneously.

### Outcomes

Delirium point-prevalence was calculated by dividing the number of patients reported with delirium by the total number of patients assessed for delirium at both 8 a.m. and 8 p.m. within each delirium literacy group.

Delirium management was appraised by evaluating the adoption of non-pharmacological interventions in accordance with the Hospital Elder Life Program (HELP) protocol [13] and identifying differences in pharmacological treatments between units/wards exhibiting high and low delirium literacy.

Additionally, the study explored qualitative aspects related to the perceived current barriers and future priorities in delirium practice and research.

### Statistical analysis

Nominal data are presented as frequency (n) and percentages (%), while metrical non-normally distributed data are described using the median and interquartile range (IQR). Comparisons based on delirium literacy were conducted using the Chi-square tests or the Fisher exact test to explore differences between groups. Statistical significance was set at the level of p < 0.05 for two-tailed tests.

The analysis was performed with R software, version 4.2.3 [23, 24].

## Results

As shown in Table 1, fifty-eight hospital wards participated in the survey, with the majority being medical/non-surgical units. Twenty-five (43.1%) wards were classified into the HL group as they fulfilled both selected criteria. Further characteristics are shown in *Supplementary Table 1s*.

**Table 1:** Characteristics of hospital wards participating in the survey: overall and by delirium literacy groups

	Overall (n = 58)	Delirium literacy	
		Low (n = 33)	High (n = 25)
Type of department/ward			
Medical/non-surgical	41 (51.7)	24 (72.7)	17 (68.0)
Surgical	14 (24.1)	6 (18.2)	8 (32.0)
Emergency Department	3 (5.2)	3 (9.1)	–
Patients age			
18–75 years	28 (48.3)	17 (51.5)	11 (44.0)
75 + years	18 (31.0)	6 (18.2)	12 (48.0)
Mixed	12 (20.7)	10 (30.3)	2 (8.0)
Number of beds in ward	25.50 [14.50, 30.00]	26.00 [17.00, 30.00]	25.00 [13.00, 32.00]

Data are shown as frequency and percentage and median and interquartile range. Delirium literacy levels were determined based on two criteria: the utilization of a validated delirium assessment tool and the presence of a written protocol for delirium management. High delirium literacy was defined by the fulfillment of both criteria

### Delirium screening, prevalence, and management

**Fig. 1:**
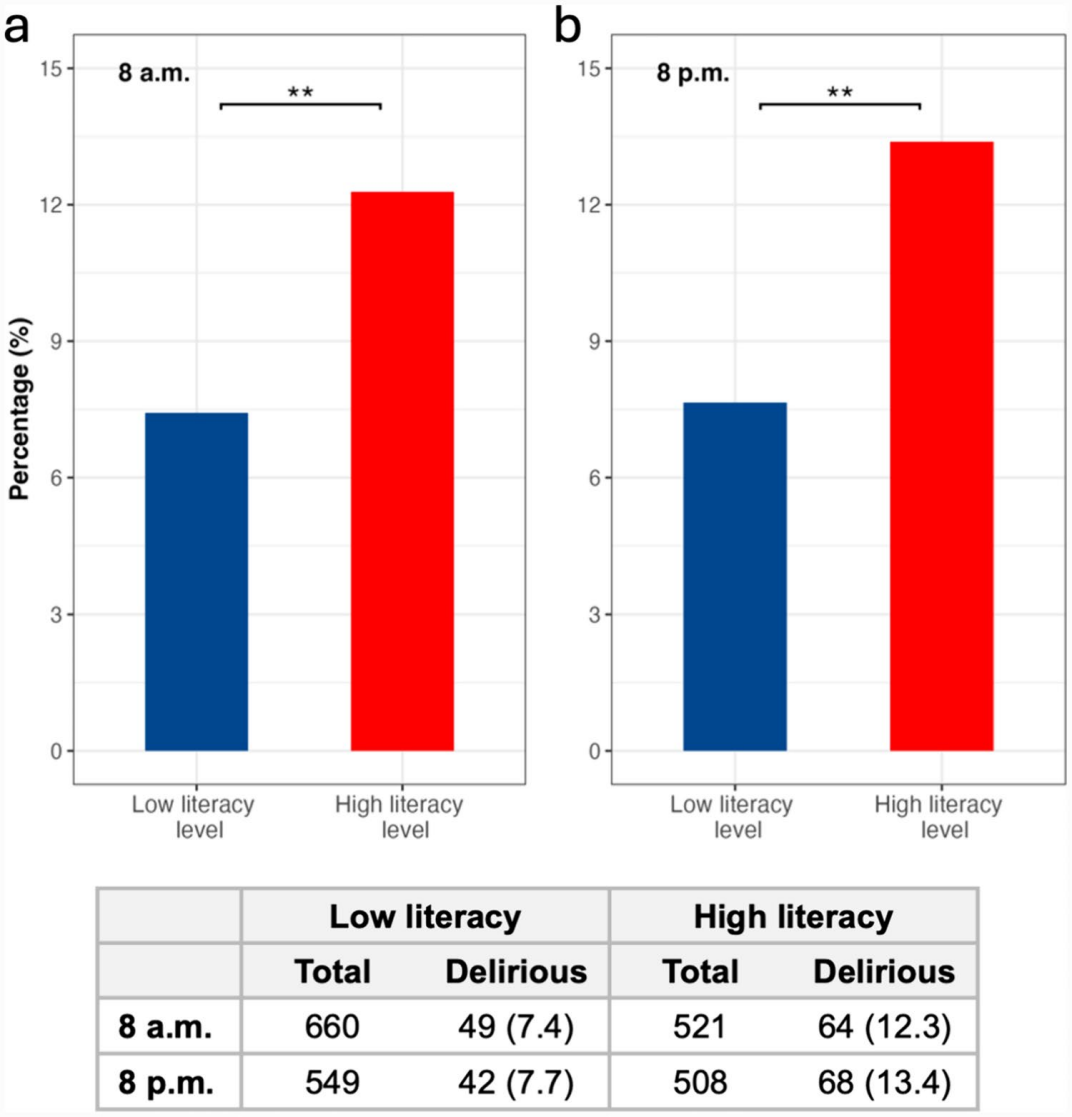
Delirium prevalence reported by survey respondents according to delirium literacy levels

Overall, the reported point prevalence of delirium was 9.6% (n = 113/1181) in the morning and 10.4% (n = 110/1057) in the evening. Notably, reported delirium prevalence was significantly higher both in the morning (12.3% vs. 7.4%, *p* = 0.006) and in the evening (13.4% vs. 7.7%, *p* = 0.003), in the HL vs. LL groups (Fig. 1).

In the HL group, the 4AT constituted the predominant delirium screening tool, used in 84.0% of cases, with the remaining using various versions of the Confusion Assessment Method (CAM). Conversely, within the low literacy group, the assessment of delirium predominantly relied on personal judgment, accounting for 60.6% of cases, followed by psychiatric consultation, Diagnostic and Statistical Manual of Mental Disorders (DSM) criteria, absence of formal tools, or other unspecified methods (see *Supplementary Fig. 1s*).

**Fig. 2:**
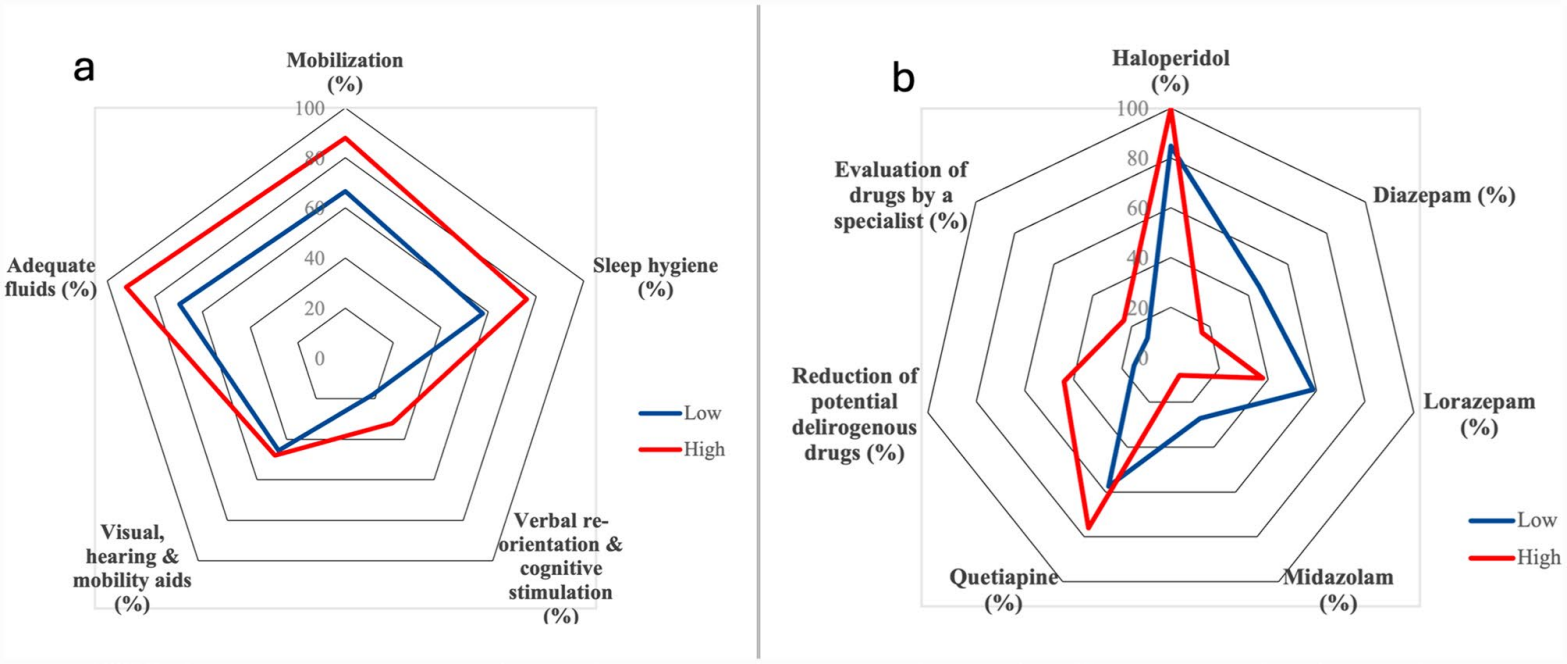
Most commonly implemented non-pharmacological and pharmacological approaches according to delirium literacy levels

In terms of delirium management, despite the lack of statistically significant differences, the HL group exhibited greater adherence to key components outlined in the HELP protocol compared to the LL group. This included higher rates of mobilization (88.0% vs. 66.7%, *p* = 0.116), sleep hygiene (76.0% vs. 57.6%, *p* = 0.237), verbal re-orientation and cognitive stimulation (32.0% vs. 18.2%, *p* = 0.364), and adequate fluid intake (92.0% vs. 69.7%, *p* = 0.080) (Fig. 2*, panel a*).

### Differences in pharmacological and non-pharmacological management

In the HL group, the most common pharmacological interventions for patients with delirium were haloperidol (100%), quetiapine (76.0%), reduction of potentially delirium-inducing drugs (44.0%), lorazepam (40.0%), specialist medication consulting (24.0%), and diazepam (16.0%). Conversely, in the LL group, the most common interventions included haloperidol (84.8%), lorazepam (54.5%), quetiapine (57.6%), diazepam (45.5%), and midazolam (27.3%) (Fig. 2, *panel b*). Significant differences between the two groups were observed for diazepam (*p* = 0.037), and the reduction of potentially delirium-inducing drugs (*p* = 0.033), which were respectively less and more prevalent in the HL group. Pharmacological management strategies in the HL group were more frequently based on standard operating procedures/protocols (56.0% vs. 6.1% in the LL group, *p* < 0.001), and individualized approaches depending on patient characteristics and side effects (80.0% vs. 42.4% in the LL group, *p* = 0.009) or delirium symptoms (60.0% vs. 24.2% in the LL group, *p* = 0.013). Additionally, recommendations for withdrawal of delirium-related drugs were reported to be more frequently included in the HL than in the LL group (44.0% vs. 15.2%, *p* = 0.033). No other significant differences emerged (see *Supplementary Table 2s*).

Further differences between the two literacy groups regarding the general management protocols enforced in the wards/units are presented in *Supplementary Table 3s.*

### Delirium-related structures and processes in the ward

Table 2 provides additional information about delirium-related structures and processes within the two groups. In the HL group, the delirium assessment was primarily conducted by physicians (56.0% vs. 24.2%), whereas in the LL group, it was carried out by unspecified mixed professionals (60.6% vs. 8.0%).

**Table 2:** Delirium-related structures and processes within the wards: overall and by delirium literacy groups

	Overall (n = 58)	Delirium literacy		p-value
		Low (n = 33)	High (n = 25)	
Healthcare professionals primarily responsible for delirium assessment				** < 0.001**
Nurse	14 (24.1)	5 (15.2)	9 (36.0)	
Physician	22 (37.9)	8 (24.2)	14 (56.0)	
Mixed professionals	22 (37.9)	20 (60.6)	2 (8.0)	
Interventions to enhance delirium awareness				
At least one educational training about delirium has been carried out in the last year	8 (13.8)	4 (12.1)	4 (16.0)	0.968
Delirium flyers	3 (5.2)	-	3 (12.0)	0.148
The term “delirium” has become commonplace in the staff’s handovers	31 (53.4)	21 (63.6)	10 (40.0)	0.128
Pocket-cards for delirium assessment/management	–	–	–	
Informational posters	–	–	–	
Delirium experts, known by the team and dedicated for delirium care, are consulted	11 (19.0)	2 (6.1)	9 (36.0)	**0.011**
Communication of delirium screening rate on the unit/ward is provided	16 (27.6)	4 (12.1)	12 (48.0)	**0.006**
None	10 (17.2)	7 (21.2)	3 (12.0)	0.569
Barriers against implementation and/or use of evidence-based strategies				
Lack of time to educate and train staff	20 (34.5)	11 (33.3)	9 (36.0)	1.000
Lack of delirium awareness	12 (20.7)	6 (18.2)	6 (24.0)	0.830
Shortage of personnel/staff	23 (39.7)	11 (33.3)	12 (48.0)	0.390
No resources for promoting delirium knowledge	9 (15.5)	5 (15.2)	4 (16.0)	1.000
Lack of delirium knowledge (i.e., treatment, assessment, etc.)	17 (29.3)	11 (33.3)	6 (24.0)	0.630
Communication gaps between professionals	9 (15.5)	7 (21.2)	2 (8.0)	0.312
Missing attitude, delirium is not important	13 (22.4)	8 (24.2)	5 (20.0)	0.948
Not enough motivated staff	5 (8.6)	2 (6.1)	3 (12.0)	0.745
Leadership does not support	1 (1.7)	–	1 (4.0)	0.888
Lack of non-pharmacological interventions to rely on	16 (27.6)	11 (33.3)	5 (20.0)	0.407
Lack of pharmacological interventions to rely on	5 (8.6)	4 (12.1)	1 (4.0)	0.536
No appropriate scores for assessment of delirium	18 (31.0)	16 (48.5)	2 (8.0)	**0.003**
Patients who are difficult for assessment (e.g. dementia, dying)	21 (36.2)	11 (33.3)	10 (40.0)	0.805
Other problems are more challenging	9 (15.5)	5 (15.2)	4 (16.0)	1.000
Inter-professional conflicts	3 (5.2)	1 (3.0)	2 (8.0)	0.804
We have no barriers; delirium is regularly assessed	6 (10.3)	1 (3.0)	5 (20.0)	0.096

Bold font indicates statistical significance

Data are shown as frequency and percentage and median and interquartile range. Multiple choices were permitted. The delirium literacy levels were determined based on two criteria: the utilization of validated assessment tools and the existence of a written protocol for delirium management. High delirium literacy was indicated by the presence of both these aspects

Regarding interventions aimed at enhancing delirium awareness, no significant differences were found in terms of educational training or the availability of informational materials. However, the presence of delirium experts (6.1% vs. 36.0%, *p* = 0.011) and the communication of delirium screening rate (12.1% vs. 48.0%, *p* = 0.006) were found to be higher in the HL group.

The most reported barriers against the implementation and/or utilization of evidence-based strategies included a shortage of personnel/staff (39.7%), difficulties in assessing complex patients (36.2%) and limited time (34.5%). No significant differences between the two groups were noted, except for the absence of an appropriate score for delirium assessment, which was more prevalent in the LL group (48.5% vs. 8.0%, *p* = 0.003).

### High-priority areas for future delirium care and research

In the analysis of free text comments regarding high-priority areas for future delirium care and research, several themes emerged, as outlined in Table 3.

**Table 3:** Survey respondents' comments regarding high-priority areas for future delirium care and research

	Categories	N° of comments	Selected example
Delirium care	Staff education	13	Conduct awareness campaigns among physicians and other professionals
	Multidisciplinary approach	3	The formation of a multidisciplinary team with advanced skills related to the topic
	Diagnostic strategies	3	The use of appropriate scores for evaluation
	Non-pharmacological management	7	Encouraging care for occupational therapy
Delirium research	Prevention	6	Correct and/or eliminate, as far as possible, the factors predisposing to delirium
	Tailored approach	1	Therapeutic approached targeted on specific groups of patients
	Pharmacological management	3	Adequate drug management
	Economic impact	1	Assessing the economic impact of non-pharmacological treatments to influence policymakers to allocate resources to the prevention of delirium

Categories were assigned based on recurring themes in the responses. The total number of responses will not sum to 58 because some survey respondents did not respond to this question, and others completed the survey multiple times for different wards while providing the same answer

Regarding delirium care, the predominant theme centered on the need for enhanced *staff education* to improve delirium care. The second core theme that emerged was *non-pharmacological management*. Respondents stressed the importance of prioritizing these strategies, such as “*encouraging care for occupational therapy”* or *“family engagement”*, as crucial priorities. Additionally, there was a call for a *multidisciplinary approach* and *diagnostic strategies* to better address delirium-related care challenges.

In terms of delirium research, the predominant theme focused on the *prevention* of delirium. Additionally, emphasis was placed on *pharmacological management*, particularly in ensuring “*adequate drug management”*. Furthermore, one respondent underscores the importance of “*assessing the economic impact of non-pharmacological treatments to influence policymakers to allocate resources to the prevention of delirium”*.

## Discussion

In this secondary analysis of data from World Delirium Awareness Day 2023 in Italian hospitals, we observed a reported delirium point prevalence of approximately 10%. Notably, the reported prevalence was two-fold higher in the HL group compared to the LL group, both in the morning and evening assessments. Moreover, the HL group demonstrated greater adherence to appropriate delirium management approaches, including both pharmacological and non-pharmacological strategies.

Previous nationwide studies conducted in older hospitalized patients reported a higher delirium point prevalence [3, 7, 9]. Several factors may contribute to the observed discrepancy in prevalence rates between our surveys and those studies. First, previous studies focused primarily on inpatients aged 65 or older, whereas this survey included a more heterogeneous age range. Given that delirium is more prevalent in older inpatients [4], this difference in age distribution could partially account for the variation in observed prevalence between studies. Moreover, this aspect could also contribute to the discrepancy in the observed prevalence of delirium between the two literacy groups, as wards/units with low literacy tended to have younger patients. Second, unlike previous studies that actively sought to detect delirium using the 4AT, this survey did not require direct assessment. Respondents were required to report the tool commonly used for delirium assessment within the ward/unit, along with the number of patients screened and identified as delirious at both time points. This variance in assessment methodology may have impacted the observed prevalence rates, as delirium tends to be underestimated without active screening [25]. Finally, this variability could partially account for the difference in reported prevalence of delirium between the HL and LL groups, since increased delirium knowledge may have facilitated more rigorous and consistent screening practices.

Another finding of our study concerns the differences in the implementation of delirium management strategies between each group. We aimed to assess the application rate of non-pharmacological approaches in accordance with the Hospital Elder Life Program (HELP) protocol [13] and of pharmacological treatments within delirium literacy groups. There was a noticeable inclination towards greater adherence in the HL versus LL group, although without a statistically significant difference. Additionally, regarding pharmacological management, the HL group demonstrated a greater attitude toward discontinuing delirium-inducing drugs and a tendency to prescribe fewer benzodiazepines. These differences suggest that specific protocols for pharmacological management within the HL group, along with increased attention to drug side effects and patient symptoms and characteristics, may have contributed to the observed trends. Furthermore, as previously demonstrated [16], the implementation of delirium management strategies has been shown to reduce delirium incidence. This could potentially explain the lower delirium prevalence observed in our survey compared to previous Italian studies.

Consistent with previous literature [19], our survey identified similar barriers against the implementation of evidence-based strategies for delirium management, which remained consistent across both literacy groups. These barriers included inadequate resources in terms of time and staff, difficulties in assessing specific patient populations (such as those with dementia), and insufficient awareness of delirium. Notably, the latter emerged as one of the key areas for future high-priority initiatives in delirium care. Furthermore, it is intricately intertwined with other priorities emphasized by the respondents, such as the use of appropriate scoring systems and the prioritization of non-pharmacological interventions. These components are essential for ensuring adequate identification and subsequent management of delirium.

In general, our findings suggest that there is still a large potential for improvement of delirium management within our country. Addressing this challenge demands the implementation of multifaceted strategies. Initiatives should commence by integrating delirium-specific training into university curricula, ensuring healthcare professionals are adequately prepared. Comprehensive awareness campaigns among healthcare personnel, ongoing professional development programs, and interdisciplinary collaboration can further enhance healthcare providers' ability to recognize, prevent, and manage delirium effectively.

Concurrently, forthcoming research on delirium should prioritize prevention strategies, foster the development of tailored approaches, and comprehensively evaluate the economic issues, both in the short- and long-term periods. This will be pivotal in influencing policymakers to allocate resources toward personnel training, preventive measures, and management strategies to overcome current barriers.

Limitations of this study include its survey design, which precluded verification of data collection and entry strategies. Participation bias may have influenced results, as clinicians with an interest in delirium were more likely to participate. Furthermore, the validity of reported delirium assessments also warrants careful consideration. Additionally, the absence of direct delirium assessment and the potential assessment by different individuals in the morning and evening could introduce bias. Finally, the exploration of delirium motor subtypes has not been conducted.

This study also exhibits several strengths, including the involvement of an interprofessional team and its nationwide scope. Moreover, conducting delirium assessment twice daily provided a more comprehensive clinical perspective. Lastly, this study may serve as a model for future quality improvement projects aimed at overcoming barriers to delirium management, thereby contributing to increased awareness about delirium.

## Conclusion

In conclusion, our secondary analysis of WDAD 2023 data provides valuable insights into current delirium care practices within Italian hospitals. Our findings emphasize the importance of enhancing awareness and implementing evidence-based strategies for delirium detection and management. These efforts are essential for optimizing delirium care, improving patient outcomes, and alleviating the burden of delirium in hospital settings.

## Supplementary Information

Below is the link to the electronic supplementary material.

Supplementary file1 (DOCX 183 KB)

## Data Availability

Data are available in anonymous form upon reasonable request addressed to the corresponding author.
