# Supplementary material for: Delirium prevalence and delirium literacy across Italian hospital wards: a secondary analysis of data from the World Delirium Awareness Day 2023
Source: Eur Geriatr Med. 2024 Jul 18;15(5):1405–13. doi: 10.1007/s41999-024-01019-5 (PMC11614987; doi:10.1007/s41999-024-01019-5)
Supplement: Supplementary file 1 — Supplementary file1 (DOCX 183 KB) [file 41999_2024_1019_MOESM1_ESM.docx]

**Delirium Prevalence and Delirium Literacy across Italian Hospital Wards: A Secondary Analysis of data from the World Delirium Awareness Day 2023**

European Geriatric Medicine

A.M. Ornago^* 1,2^, E. Pinardi^* 1,2^, C. Okoye^1,2,3^, P. Mazzola^1,3^, M.C. Ferrara^1^, A. Finazzi^1^, P. Nydahl^4,5^, R. von Haken^6^, H. Lindroth^7,8^, K. Liu^9,10^, A. Morandi^§^ ^11,12^, G. Bellelli^§^ ^1,3^

**Affiliations:**

^1^ School of Medicine and Surgery, University of Milano-Bicocca, Milan, Italy

^2^ Aging Research Center, Department of Neurobiology, Care Sciences and Society, Karolinska Institutet and Stockholm University, Stockholm, Sweden

^3^ Acute Geriatric Unit, IRCCS San Gerardo Foundation, Monza, Italy

^4^ Nursing Research, University Hospital Schleswig-Holstein, Kiel Germany

^5^ Institute of Nursing Science and Development, Paracelsus Medical University, Salzburg, Austria

^6^ Department of Anesthesiology, University Hospital Mannheim, Germany

^7^ Division of Nursing Research, Department of Nursing, Mayo Clinic, Rochester, MN, USA

^8^ Center for Aging Research, Regenstrief Institute, School of Medicine, Indiana University, Indianapolis, IN, USA

^9^ Critical Care Research Group, The Prince Charles Hospital, Brisbane, Australia

^10^ Radboud University Medica Center, Department Intensive Care, Nijmegen, the Netherlands

^11^ Intermediate Care and Rehabilitation, Azienda Speciale Cremona Solidale, Cremona, Italy

^12^ Parc Sanitari Pere Virgili, Val d’Hebron Institute of Research, Barcelona, Spain

* A.M. Ornago and E. Pinardi are co-first author

§ G. Bellelli and A. Morandi are co-senior author

**Keywords**: delirium; hospital; survey; quality improvement

**Running title**: Delirium in Italian hospital wards on WDAD 2023

**Corresponding author**:

Alice Margherita Ornago

School of Medicine and Surgery, University of Milano-Bicocca

Piazza dell’Ateneo Nuovo 1, Milan, Italy

E-mail address: [a.ornago@campus.unimib.it](mailto:a.ornago@campus.unimib.it)

ORCID: orcid.org/0009-0008-7927-793X

**SUPPLEMENTARY MATERIALS**

**Fig.1s Tools used for assessing delirium in the high and low literacy groups**


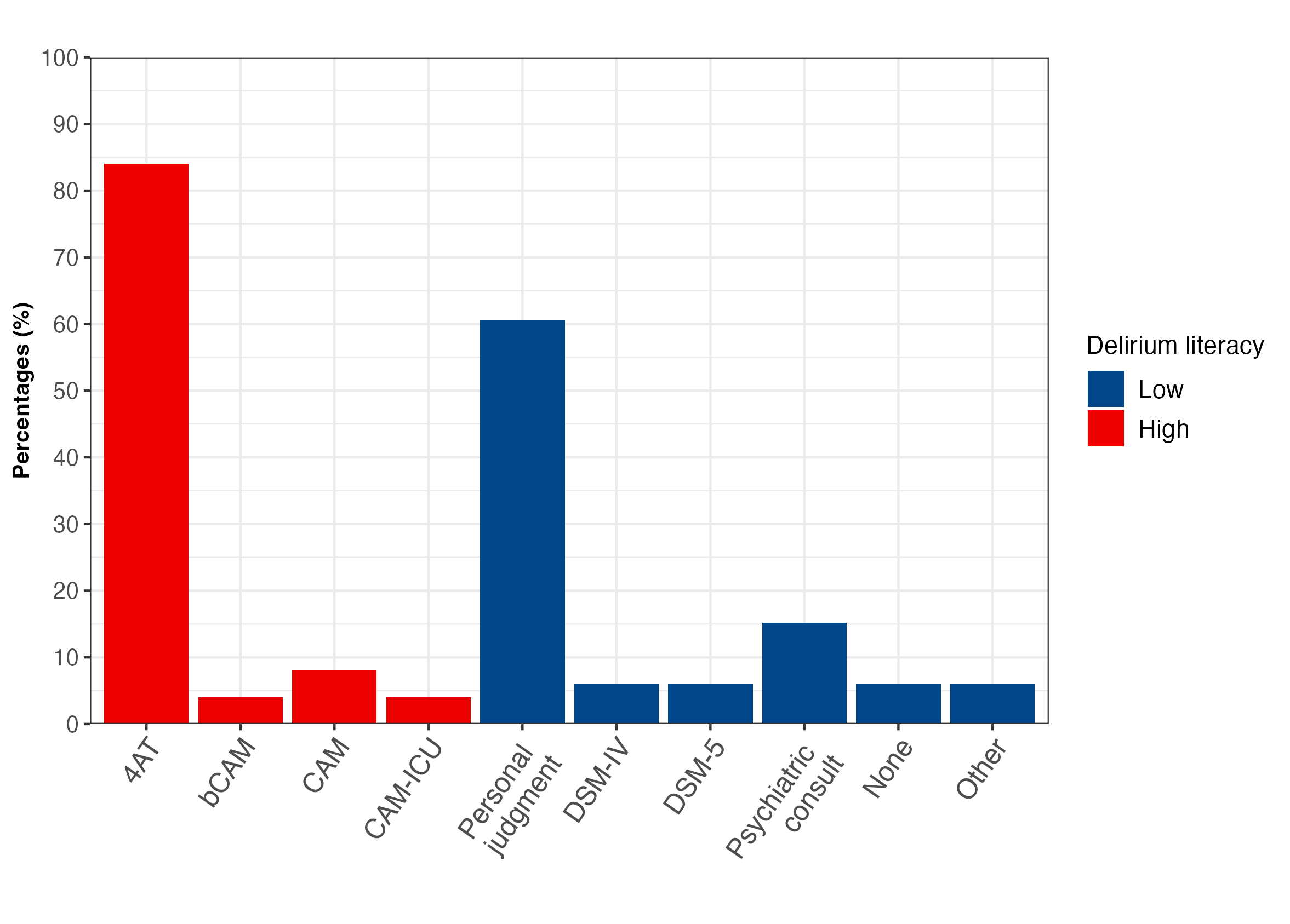


**Table 1s. Characteristics of hospital surveys**

|  |  | **Delirium literacy** | |  |
| --- | --- | --- | --- | --- |
|  | **Overall**  **(n = 58)** | **Low**  **(n= 33)** | **High**  **(n= 25)** | **p-value** |
| **Hospital geographic area** |  |  |  | **<0.001** |
| Northern Italy | 35 (60.3) | 26 (78.8) | 9 (36.0) |  |
| Central Italy | 18 (31.0) | 4 (12.1) | 14 (56.0) |  |
| Southern Italy and Islands | 5 (8.6) | 3 (9.1) | 2 (8.0) |  |
| **Hospital type** |  |  |  | 0.270 |
| University hospital | 44 (75.9) | 22 (66.7) | 22 (88.0) |  |
| University related/affiliated hospital | 7 (12.1) | 5 (15.2) | 2 (8.0) |  |
| Community hospital | 6 (10.3) | 5 (15.2) | 1 (4.0) |  |
| Others | 1 (1.7) | 1 (3.0) | - |  |
| **Survey’s Respondents Profession** |  |  |  | **<0.001** |
| Physician | 35 (60.3) | 11 (33.3) | 24 (96.0) |  |
| Nurse | 23 (39.7) | 22 (66.7) | 1 (4.0) |  |

**Table 2s. Pharmacological management: overall and by delirium literacy groups**

|  |  | **Delirium literacy** | |  |
| --- | --- | --- | --- | --- |
|  | **Overall**  **(N = 58)** | **Low**  **(N = 33)** | **High**  **(n = 25)** |  |
| **The majority (>50%) of patients with delirium receive** |  |  |  |  |
| Haloperidol | 53 (91.4) | 28 (84.8) | 25 (100.0) | 0.118 |
| Clonidine | 3 (5.2) | 3 (9.1) | - | 0.342 |
| Melperone | - | - | - | - |
| Risperidone | 8 (13.8) | 4 (12.1) | 4 (16.0) | 0.968 |
| Lorazepam | 28 (48.3) | 18 (54.5) | 10 (40.0) | 0.405 |
| Dexmedetomidine | 2 (3.4) | 2 (6.1) | - | 0.599 |
| Diazepam | 19 (32.8) | 15 (45.5) | 4 (16.0) | **0.037** |
| Quetiapine | 38 (65.5) | 19 (57.6) | 19 (76.0) | 0.237 |
| Midazolam | 11 (19.0) | 9 (27.3) | 2 (8.0) | 0.130 |
| Distraneurin | - | - | - | - |
| Melatonin | 7 (12.1) | 6 (18.2) | 1 (4.0) | 0.217 |
| Beta-blocker | 1 (1.7) | - | 1 (4.0) | 0.888 |
| Levodopa | 1 (1.7) | 1 (3.0) | - | 1.000 |
| Phenobarbital | 4 (6.9) | 4 (12.1) | - | 0.200 |
| Reduction of potentially delirium-inducing drugs | 16 (27.6) | 5 (15.2) | 11 (44.0) | **0.033** |
| Evaluation of drugs by a specialist (e.g. geriatrician, pharmacists, or else) | 10 (17.2) | 4 (12.1) | 6 (24.0) | 0.404 |
| Do not know | 1 (1.7) | 1 (3.0) | - | 1.000 |
| Trazodone | 7 (12.1) | 4 (12.1) | 3 (12.0) | 1.000 |
| Promazine | 9 (15.5) | 5 (15.2) | 4 (16.0) | 1.000 |
| Delorazepam | 4 (6.9) | 2 (6.1) | 2 (8.0) | 1.000 |
| **The pharmacological management of delirium patients** |  |  |  |  |
| Is based on a standard operation procedure (SOP), or protocol | 16 (27.6) | 2 (6.1) | 14 (56.0) | **<0.001** |
| Includes pharmacologist’s support | 2 (3.4) | 1 (3.0) | 1 (4.0) | 1.000 |
| Includes psychiatrist or delirium-specific liaison team | 25 (43.1) | 16 (48.5) | 9 (36.0) | 0.495 |
| Is a more general approach, including a few pharmacological agents | 16 (27.6) | 6 (18.2) | 10 (40.0) | 0.122 |
| Is a more individual approach, depending on patients, and side effects | 34 (58.6) | 14 (42.4) | 20 (80.0) | **0.009** |
| Depends on specific symptoms of each patient’s delirium | 23 (39.7) | 8 (24.2) | 15 (60.0) | **0.013** |
| Is discussed with patients in most cases | 1 (1.7) | 1 (3.0) | - | 1.000 |
| Is discussed with families in most cases | 14 (24.1) | 7 (21.2) | 7 (28.0) | 0.773 |
| Is reported in handovers | 23 (39.7) | 16 (48.5) | 7 (28.0) | 0.191 |
| Includes recommendations for withdrawal of delirium-related drugs | 16 (27.6) | 5 (15.2) | 11 (44.0) | **0.033** |
| None of the above | - | - | - | - |
| Other | - | - | - | - |

Data are shown as frequency and percentage. Multiple choices were permitted. The delirium literacy levels were determined based on two criteria: the utilization of validated assessment tools and the existence of a written protocol for delirium management. High delirium literacy was indicated by the presence of both these aspects.

**Table 3s. Written protocols: overall and by delirium literacy groups**

|  |  | **Delirium literacy** | |  |
| --- | --- | --- | --- | --- |
| **Presence of written protocols within the ward** | **Overall**  **(n= 58)** | **Low**  **(n= 33)** | **High**  **(n= 25)** | **p-value** |
| Pain management (assess, prevent, and manage pain) | 43 (74.1) | 21 (63.6) | 22 (88.0) | 0.073 |
| Spontaneous Awakening Trial (SAT) management | 11 (19.0) | - | 11 (44.0) | **<0.001** |
| Spontaneous breathing trial (SBT) management | 12 (20.7) | 1 (3.0) | 11 (44.0) | **<0.001** |
| Sedation management | 20 (34.5) | 4 (12.1) | 16 (64.0) | **<0.001** |
| *Delirium management* | 30 (51.7) | 5 (15.2) | 25 (100.0) | **<0.001** |
| Dementia | 17 (29.3) | 2 (6.1) | 15 (60.0) | **<0.001** |
| Mobility and exercise | 23 (39.7) | 10 (30.3) | 13 (52.0) | 0.161 |
| Family engagement and empowerment | 31 (53.4) | 12 (36.4) | 19 (76.0) | **0.006** |
| Nutrition management | 27 (46.6) | 11 (33.3) | 16 (64.0) | **0.040** |
| Sleep | 4 (6.9) | 2 (6.1) | 2 (8.0) | 1.000 |
| Physical restraint | 39 (67.2) | 16 (48.5) | 23 (92.0) | **0.001** |
| ICU Diaries | 18 (31.0) | 4 (12.1) | 14 (56.0) | **0.001** |
| None of the above | 2 (3.4) | 2 (6.1) | - | 0.599 |
| Other | 7 (12.1) | 4 (12.1) | 3 (12.0) | 1.000 |

Data are shown as frequency and percentage. Multiple choices were permitted. The delirium literacy levels were determined based on two criteria: the utilization of validated assessment tools and the existence of a written protocol for delirium management. High delirium literacy was indicated by the presence of both these aspects.
